# Pain in people with dementia is a significant contributor to increased professional caregiver burden: results of an observation study

**DOI:** 10.3389/fpain.2026.1868192

**Published:** 2026-07-30

**Authors:** Kreshnik Hoti, Ipsit Vahia, Paola Chivers, Jeffery Hughes

**Affiliations:** 1 Faculty of Medicine, University of Prishtina, Prishtina, Kosovo; 2 McLean Hospital, Belmont, MA, United States; 3 Harvard Medical School, Boston, MA, United States; 4 Institute for Health Research, The University of Notre Dame Australia, Fremantle, WA, Australia; 5 School of Medical and Health Sciences, Edith Cowan University, Perth, WA, Australia; 6 Research Department, Child and Adolescent Health Service, Perth, WA, Australia; 7 School of Diagnostic and Therapeutic Studies, Curtin University, Perth, WA, Australia; 8 PainChek Ltd, Sydney, NSW, Australia

**Keywords:** caregiver burden, caregiver score, dementia, neuropsychiatric symptoms, pain

## Abstract

**Aim:**

To investigate the association between the presence of neuropsychological symptoms, professional caregiver burden and pain intensity.

**Methods:**

Eleven neuropsychiatric symptoms reportedly associated with caregiver burden which are included within the PainChek® pain assessment tool were identified: aggressiveness, inappropriate behavior, distressed, verbally offensive, confusion, restlessness, pacing and wandering, crying, screaming, altered sleep and resisting care. A caregiver burden score based on binary scoring of the presence of these indicators was calculated (summative score range from 0 to 11). Generalized estimating equations were used to evaluate the relationships between (1) pain score and sum of caregiver indicators present and (2) pain (high/low) and caregiver burden indicator presence.

**Results:**

The results of 2,125,022 PainChek® assessments completed on 89,230 residents (Average age 84.8 ± 7.7 years, females 62.2%) were evaluated. The binary pain model reported a significant increased risk of high (i.e., moderate or severe) pain (*p* < 0.001) for all caregiver burden indicators except pacing and wandering (*p* = 0.494). Being distressed was associated with the highest risk (OR = 12.0) of high pain, followed by altered sleep (OR = 6.0). Age (*p* = 0.004) and sex (*p* < 0.001) were both significant confounders, with high pain less likely with increasing age and if a patient was female.

**Conclusions:**

Pain associated neuropsychiatric symptoms can contribute to professional caregiver burden and are exacerbated by high pain, emphasizing the need to minimize pain in people living with dementia.

## Introduction

Symptoms of dementia affect people in a way that diminishes their ability to function independently, therefore increasing their dependency, need for caregiving and support of daily living activities by people directly involved in provision of care i.e., caregivers (1). This can create caregiver burden which is the strain that is experienced by the caregiver looking after the person who is either ill, disabled or elderly (2, 3). Whilst caregiver burden is often experienced by family members (often referred to as informal caregivers), it is also an issue for healthcare professionals and other carers (e.g., personal care assistants) as professional caregivers or formal caregivers (1, 4).

The negative consequences of caregiver burden can impact both the person providing the care and the care recipient. These negative consequences include decreased provision of care, reduction in quality of life of both care recipient and caregiver, and deterioration of physical health of the caregivers leading to fatigue and chronic diseases, together with negatively impacting caregivers' psychological health resulting stress and anxiety (3, 5–8). It has been reported that when caregivers experience caregiver burden in the absence of adequate support or resources, the quality of care they provide is reduced (9, 10). This may arise because of a decreased ability of the caregiver to cope with the demands of delivering care or as a result of burnout where the caregiver can no longer provide emotional support for the person they care for (9, 10).

In people living with dementia, caregiver burden is complex. In addition to delivery of care being time consuming, there is a substantial financial burden as well (7, 8). Caregiver burden is affected by a number of factors related to the caregiving process, the contextual factors as well as those related to the person living with dementia themselves, such as their socio-demographic features (11, 12). In addition to type of dementia and degree of impairment, clinical characteristics of people with dementia have also been reported as contributors to this burden. In this regard, presence of neuropsychiatric symptoms of dementia are important considerations (13–17) and have been linked to a higher burden of care regardless of the degree of cognitive decline or its etiology (15). A relationship has been established between higher caregiver burden and neuropsychiatric symptoms, especially challenging behaviors (also referred to as responsive behaviors) in people living with dementia (14, 18). In the case of those caring for people with dementia, neuropsychiatric symptoms can be significant barriers to care provision and are often perceived as an inevitable consequence of disease progression, when in fact they may arise from treatable causes such as pain.

Caregiver burden can be managed with specific interventions aimed at reducing either the psychological or non-psychological components of burden, or both (3, 8). However, evidence is lacking on the relationship between various pain related manifestations and caregiver burden in people living with dementia. A better understanding of this could provide more insight into how pain related interventions can affect caregiver burden and hence inform potential mitigation strategies. In this study we aimed to examine the relationship between pain and caregiver burden, as seen by pain related features experienced by residents living with dementia. In this regard, we specifically aimed to identify various neuropsychiatric symptoms manifested through pain, which were collected digitally at the point-of-care, that can contribute to caregiver burden. In doing so, our objective was to demonstrate how managing pain and positively impacting pain-related neuropsychiatric symptoms are important to consider when designing interventions that deal with patient-experienced behavioral components of professional caregiver burden.

## Methods

This is an observational study in which we reviewed a retrospective database of 2,125,022 pain assessments conducted in 89,230 nursing home residents unable to reliably self-report pain. These assessments were conducted over a period of 5.3 years (i.e., form 15 February 2018 to 14 June 2023) in multiple nursing homes around Australia, New Zealand and the United Kingdom (UK). Pain assessments were conducted using the PainChek® pain assessment tool by a total of 14,987 assessors from 434 different providers. Assessors were nursing home staff who received 2-hours training on the use of this tool as well as general training on common triggers and challenges of pain assessment in people living with dementia.

The PainChek® pain assessment system consists of three key components: the pain assessment tool (i.e., the mobile app), digital analytics and education components. This tool is a digital point-of-care solution which consists of 6 domains of pain assessment containing a total of 42 pain indicators: Face (which uses Artificial Intelligence to detect 9 facial action units indicative of pain), Voice (9 pain indicators), Movement (7 pain indicators), Behavior (7 pain indicators), Activity (4 pain indicators) and Body (6 pain indicators). Non-facial indicators of pain are collected through a digital checklist. A binary scoring system for each pain indicator, through a smart automation process allows generation of pain score and intensity categorized as follows: no pain (0–6), mild pain (7–11), moderate pain (12–15) and severe pain (16–42). Further details on the PainChek® pain assessment tool as well as its validation have been published elsewhere (19–23).

The initial ethics approval for this study was granted from the Human Research Ethics Committee of Curtin University (HR10/2014), subsequently by Ethics Committee of the Faculty of Medicine, University of Prishtina (10941/2023).

PainChek Ltd granted permission to access the dataset used in this study. The anonymized data was compiled from pain assessments conducted and synchronized into web administration portal (PainChek® portal). These assessments were conducted at the point-of-care using the PainChek® pain assessment tool as part of routine clinical practice. All data accessed is deidentified and made available by PainChek Ltd in accordance with its service agreement with aged care providers. Care providers have separated agreements with their clients and legally authorized representatives regards use of their de-identified clinical data.

### Defining caregiver burden items in the PainChek® pain assessment tool

**Figure 1:**
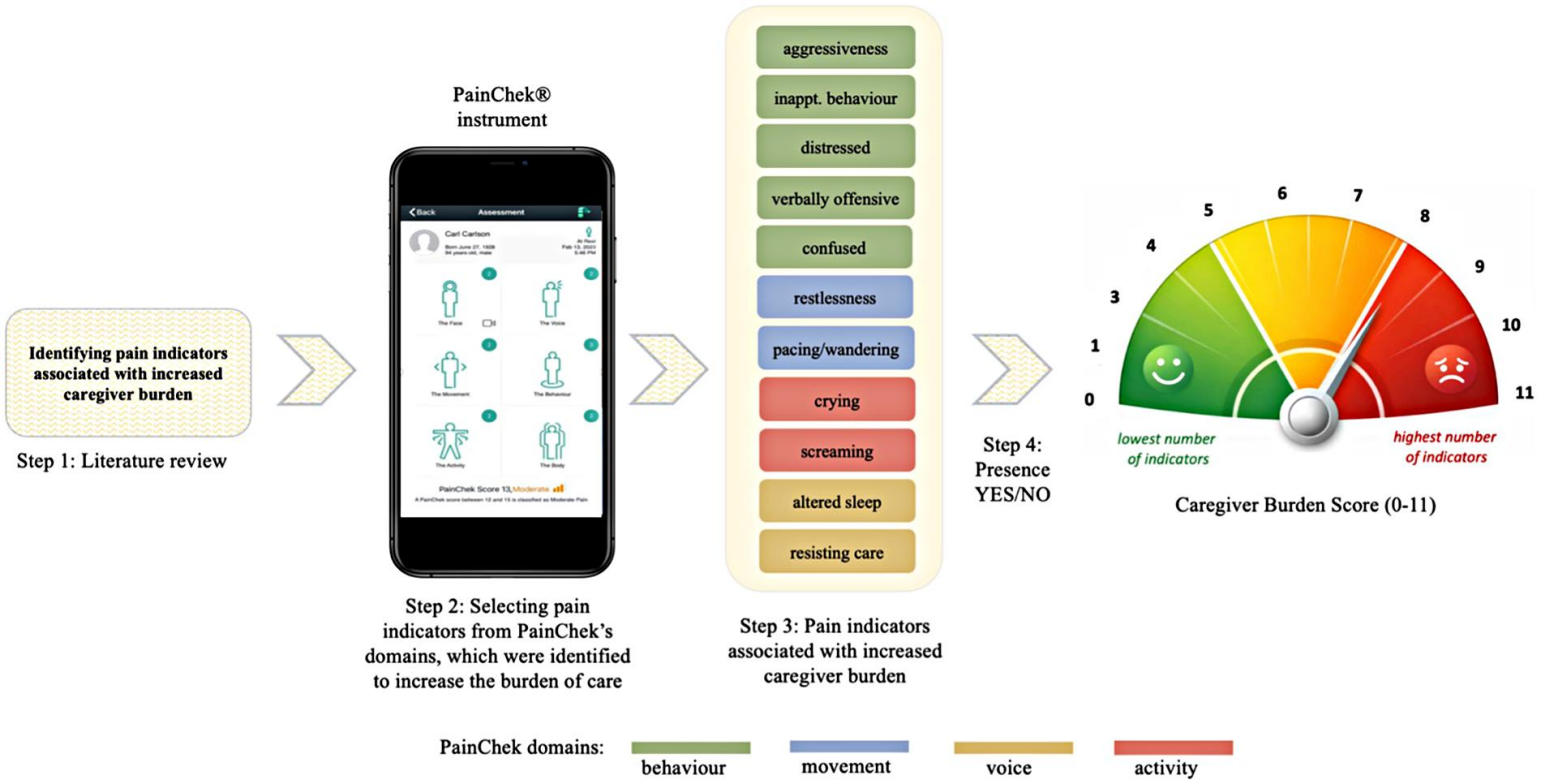
Selection process for pain indicators associated with an increase in caregiver burden and creation of a caregiver burden score.

A literature review was undertaken to identify pain related indicators included within the PainChek® pain assessment instrument, which are reported (as per our literature review) to contribute to increased caregiver burden (Figure 1). The following databases were searched: MEDLINE, Embase PubMed and Joanna Briggs Institute Database from inception to 31st of August 2025. We searched for the terms: carer or caregiver, burden, dementia, behaviors, neuropsychiatric symptoms, behaviors and dementia in searching databases. This review identified 11 PainChek® pain indicators which are reported to increase caregiver burden.

For the purpose of this study, a caregiver burden score was calculated by summing identified pain indicators that increase the burden of care that are present in non-facial domains of the PainChek® pain assessment tool. The same weighing was assigned to each pain indicator. A binary scoring system was used with indicators either scored as present (1) or absent (0), with the sum score range being 0–11 (Figure 1). Following this process, a caregiver score ranges from 0 to 11 with 11 representing the highest burden of care.

#### Data analysis

Caregiver burden scores were described using mean (M), standard deviation (SD), median (Md) and interquartile range (IQR). The relationship with pain score was depicted using a violin plot. Negative binomial (log link) Generalized Estimating Equation (GEE) model examined the predictive contribution of each of caregiver burden (covariate) on pain score with confounders age and sex, all treated as fixed effects, and patients treated as repeated measures. A binary logistic (GEE) model was used for comparing caregiver burden indicators with outcome pain intensity: low pain=no pain or low pain; high pain=moderate or severe. Model fixed effect Wald *χ*^2^ and *p*-values, beta coefficient (*β*) and standard error (SE) for age, odds ratio [Exp (*β*)] for sex and indicators, and 95% confidence intervals (CIs) are reported.

## Results

Of the 89,230 residents in whom pain assessments were conducted, residents' age was between 65 and 110 years (M = 84.8, SD = 7.7, 95%, Md = 85.0, IQR 80–91) with almost two-thirds being females (55,499, 62.2%). Over half of the total number of residents (i.e., 52.8%) experienced at least one episode of pain (i.e., PainChek® score >6). Most assessments were conducted in Australian nursing homes (1,981,745 93.3%), followed by the UK (134,807 6.3%), with a small proportion contributed from New Zealand. Pain assessments were conducted by 14,987 trained assessors from 434 different providers. Typically, PainChek® users are professional caregivers including registered nurses (RN), enrolled nurses (EN), personal care assistants (PCA) or “dementia consultants” [i.e., a senior Registered Nurse (RN), often holding a title such as a Clinical Nurse Consultant (CNC) or Specialized Dementia Advisor], with a smaller number of allied health professionals (24).

Pain related indicators present in the PainChek® pain assessment tool were identified from the literature review to increase the caregiver burden were: aggressiveness (PainChek' Behavior domain), inappropriate behavior, distressed (Behavior domain), verbally offensive (Behavior domain), confused (Behavior domain); restlessness (Activity domain), pacing/wandering (Activity domain), crying (Voice domain), screaming (Voice domain), altered sleep patterns (Activity domain) and resisting care (Activity domain). The selection process of the above 11 pain indicators associated with caregiver burden and therefore establishment of the caregiver score range between 0 and 11 is illustrated in Figure 1.

Caregiver burden score association with pain intensity category is shown in Table 1. Increasing intensity of pain was associated with increased caregiver burden score. This was evident incrementally when pain is categorized as low or high or when pain is categorized as no pain (PainChek® score = 0–6), mild pain (7–11), moderate pain (12–15) and severe pain (16–42).

**Table 1:** Caregiver burden score for total sample and pain groups.

Pain Group	Pain Level	Caregiver Burden Score	
		M (SD)	Md (IQR)
Total	All Pain	0.8 (1.3)	0.0 (0.0–1.0)
Pain 2-group	Low Pain	0.7 (1.1)	0.0 (0.0–1.0)
	High Pain	4.3 (2.0)	4.0 (3.0–6.0)
Pain 4-group	No pain	0.6 (0.9)	0.0 (0.0–1.0)
	Mild pain	2.5 (1.5)	2.0 (1.0–3.0)
	Moderate pain	3.9 (1.8)	4.0 (3.0–5.0)
	Severe pain	5.3 (2.0)	5.0 (4.0–7.0)

M, mean, SD, standard deviation, Md, median, IQR, interquartile range (25th – 75th percentile). Low pain comprises no and mild pain, high pain comprises moderate and severe pain categories.

**Figure 2:**
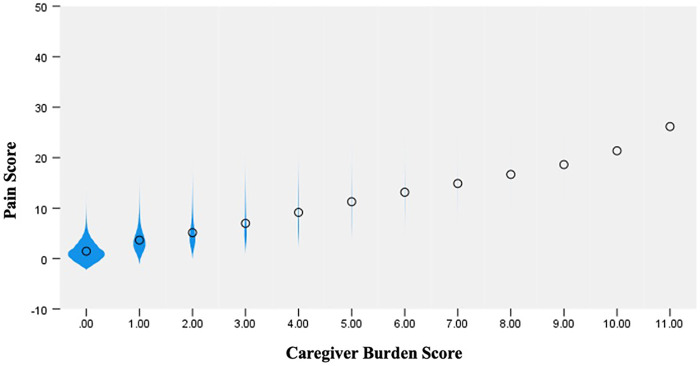
Association between caregiver burden score (0−11) and PainChek® pain score (0–42). Violin plots represent the distribution of caregiver burden scores at each pain score.

The association between residents' pain scores and caregiver burden raw scores is depicted in Figure 2. Negative binomial GEE reported that residents' pain scores were positively associated with the number of caregiver burden indicators present (Wald *χ*^2^ = 39605.0; *p* < 0.001) with a significant confounding by for age (Wald *χ*^2^ = 9.9 p. = 0.002) but not sex (Wald *χ*^2^ = 0.9 *p* = 0.345).

For the caregiver burden binary logistic GEE model, pain indicators present were each significantly associated with increased odds of high pain (*p* < 0.001) except for the pacing/wandering pain indicator present in PainChek's “Movement” domain of pain indicators (*p* = 0.494). The presence of the pain indicator “distressed” contained in the PainChek® “Behavior” domain, was associated with the highest risk of high pain (OR = 12.0), suggesting its highest potential predictability related to caregiver burden as well. This was followed by altered sleep (OR = 6.0), which is a pain indicator present in the PainChek® “Activity” domain, crying (OR = 4.4) in the “Voice” domain and restlessness (OR = 4.2) present in the “Movement” domain. On the other hand, pain indicator “confused” (OR = 1.6) present in the “Behavior” domain, was associated with the lowest odds of high pain. (Table 2) In this regard, both age and sex were significant confounders (*p* < 0.001).

**Table 2:** Generalized estimating equation (GEE) predictability of high pain scores related to the presence of individual caregiver burden indicators.

Caregiver Burden Indicator†	Pain assessment domain in PainChek® pain assessment tool		95% CI Exp (*β*)			
		Exp (β)	Lower	Upper	*χ* ^2^	*p*-value
Age (years)		1.0	1.0	1.0	8.4	0.004
Sex female^a^		0.9	0.8	0.9	12.3	<0.001
Aggressive^b^	Behavior	2.4	2.3	2.6	770.8	<0.001*
Inappropriate behavior^b^	Behavior	3.0	2.8	3.2	1,100.3	<0.001*
Restlessness^b^	Movement	4.2	4.0	4.4	3,299.6	<0.001*
Distressed^b^	Behavior	12.0	11.4	12.6	9,160.5	<0.001*
Verbally offensive^b^	Behavior	2.8	2.6	3.0	804.5	<0.001*
Confused^b^	Behavior	1.6	1.5	1.7	246.6	<0.001*
Pacing/wandering^b^	Movement	1.0	0.9	1.1	0.5	0.494
Crying^b^	Voice	4.4	4.1	4.8	1,272.4	<0.001*
Altered sleep^b^	Activity	6.0	5.6	6.4	2,933.8	<0.001*
Resisting care^b^	Activity	3.7	3.5	3.9	2,647.3	<0.001*
Screaming^b^	Voice	2.2	2.0	2.3	373.1	<0.001*

**^†^**Burden of care indicator present in the PainChek® instrument. Pain(low/high) model reports a binary logistic with Odds Ratio [Exp(*β*)] and 95% confidence intervals (CI) presented; Category compared to ^a^male ^b^absent.

*Statistically signiﬁcant at *p* *<* 0.000001.

## Discussion

We report our findings from exploring a digitally collected large database of pain assessments conducted at the point-of-care. Here we analyzed a total of 11 different neuropsychiatric pain indicators, present in the PainChek® pain assessment tool, which we identified from literature review to be associated with increasing the burden of care. These pain indicators were used to calculate a caregiver burden score. Our findings suggest a clear positive relationship between pain and caregiver burden score, based on pain assessments completed by professional caregivers. In this regard, higher levels of pain were associated with an increase in caregiver burden score. This was evident incrementally throughout different levels of pain intensity, from mild to severe. This is a clinically relevant finding considering that caregiver strain impacts provision of care and their capacity to monitor the resident living with dementia whereas when these residents experience higher pain levels their caregiver needs may increase. This is also supported by findings of Tsuji et al. (2022) who reported a correlation between caregiver burden and pain intensity (25). In the abovementioned association between individual caregiver burden indicators and high pain, age was a significant confounder but not gender. In comparison, previous research has suggested no significant difference in relation to caregiver burden and care recipient's age (1, 26). This difference may be related to the fact that these authors measured perceived caregiver burden using the Zarit Burden Interview (ZBI), which is caregiver centric, whereas in our study we explored specific resident experienced symptoms, which are neuropsychiatric in nature, to calculate the caregiver burden score. Importantly, in study by Tsai et al, using the neuropsychiatric inventory, which calculates the presence and severity of neuropsychiatric symptoms, there was a significant association with ZBI score and caregiver burden in relation to age but not gender, hence confirming our findings (1).

Our study also showed that, with the exception of pacing and wandering, each caregiver burden related pain indicator was associated with increased odds of high pain levels (i.e., moderate and severe levels of pain). Exception of pacing/wandering to this relationship is an interesting finding and may be explained by the fact that although this is an important pain indicator included in multiple pain assessment tools to increase the sensitivity of pain detection, multiple potential etiologies rather than nociception alone could be involved. Additionally, Sampson et al. (2015) found a lack of association between pain and symptoms related to activity disturbance which do not require ambulation whereas Ahn and Horgas (2013) suggested that residents with severe pain are less likely to experience wandering behaviors (27, 28). The later especially, is in line with our findings. Overall, the association with increased odds of most neuropsychiatric pain indicators with higher pain suggests that, during pain management, caregiver burden reduction can be effectively ensured through monitoring of patient related neuropsychiatric pain indicators. This could provide a more direct and patient-centric approach to measuring caregiver burden and its reduction during pain management.

Our findings suggest that the pain indicator “distressed” is associated with the highest risk of the presence of high pain and therefore it could have the highest potential for predicting caregiver burden as well. This was followed by altered sleep. This is in line with literature that supports the relationship of these symptoms with increasing caregiver burden (29). This has important clinical implications, especially considering that distress behaviors can arise from unmet needs of people living with dementia (30). Where these behaviors are related to pain they can be reduced through improved pain management, resulting in better quality of care for people living with dementia (27). In this regard, studies have shown that addressing pain leads to a reduction in symptoms such as distress and sleep disturbances (31, 32). In general, a better understanding of not just the relationship of the caregiver burden score with pain but identifying specific neuropsychiatric symptoms such as distress and altered sleep can enable better monitoring and evaluation of their impact on the caregiver burden.

A key strength of this study relates to its representativeness considering that its findings come from what is to our knowledge world's largest database of pain assessments conducted across multiple nursing home sites as well a number of countries. Further, these pain assessments were conducted by thousands of assessors who received specific training. This ensures a high degree of standardization in terms of pain assessments conducted. Further, collection of caregiver burden related indicators through a digital solution ensured a fast and reliable documentation at the point-of-care. Additionally, we evaluate the relationship between caregiver burden and pain through resident experienced neuropsychiatric indicators. This provides further patient-centric insights, in comparison to exploring the burden of care from perceived perspectives of caregivers only (1, 26). Our study has a number of limitations as well. Firstly, we only explored 11 pain indicators present in the PainChek® pain assessment tool which are associated with burden of care, out of a total of 42 pain indicators present in this tool. Importantly, these burden of care predictors are derived from items embedded with the PainChek® assessment itself, introducing a degree of shared measurement structure. This may result in potential inflation of observed associations between indicator presence and pain scores. Accordingly, these relationships should be interpreted with caution. Notwithstanding this limitation, the analysis provides clinically relevant insight into how pain-related neuropsychiatric features observed at the point-of-care may concurrently reflect both pain experience and elements contributing to caregiver burden. There are also other predictors of caregiver burden and hence our findings should be interpreted taking that into consideration (1, 12). Additionally, these indicators are assessed on a binary scale (present vs. absent) as opposed to individual severity of their presence, which may have allowed a more granular understanding of the indicator. Nonetheless, the binary approach ensures standardization during assessment and reduction of observer bias which may arise from assessors assigning a perceived level of indicator severity. Secondly, analysis of a data collected did not allow us to compare resident experienced neuropsychiatric indicators with the actual perceived burden of care by caregivers involved.

Thirdly, we did not account for dementia sub-type and its severity, which were not available in the dataset. These are important considerations considering that pain processing can be affected by different types of dementia (33, 34). Nonetheless our findings highlight the importance of resolving pain in people living with dementia, which can have relevant implications for caregiver burden as well.

Fourthly, we did not have access to the residents preexisting or comorbid conditions, as this information is not captured in the PainChek® database, this is important as certain conditions may directly cause distress symptoms which are captured in the PainChek® pain assessment tool, but are not pain related (e.g., confusion associated with a urinary tract infection). This is important to consider when interpreting our results. However, the comprehensive nature of the pain indicators included PainChek® pain assessment tool aims to reduce the risk of misinterpreting non-pain associated distress with pain related distress. Sampson et al. (2025) found a significant difference between pain assessment conducted at rest and post-movement. Based on this finding they commented their results 'suggests that PainChek® may be useful in distinguishing between pain and discomfort due to non-pain (non-nociceptive) causes.' (35) Differences in pain levels between rest and post-movement were also confirmed during pain assessments conducted with PainChek® in real-world use (36).

Fifthly, we did not consider the possible impact of the use of medications, such as opioids, antipsychotics and sedatives on residents' symptoms, as this data is not available within the PainChek® database. However, we would argue if neuropsychiatric symptoms persisted despite the use of such medications, that these residual symptoms can continue to contribute to caregiver burden, as mentioned above.

Lastly, in this study all pain assessments were completed by professional caregivers and the people living with dementia were all in residential care, which raises the question of generalizability of our findings to family members and other carers looking after people with dementia in the community. However, the 11 pain indicators chosen based on our literature review are said to be applicable to professional (formal) and family (informal) caregivers, although the importance of each may vary. For example, Seidel and Thyrian (2017) reported that family caregivers perceived cognitive impairment, aggressive and disoriented behavior of the person with dementia as greater contributors to caregiver burden than their professional caregiver counterparts (37). Notwithstanding this, pain is common amongst community dwelling people with dementia as it is with people living in residential care, suggesting the findings should be applicable to both settings (38, 39).

Further research is needed to validate our proposed caregiver burden score against professional caregiver burden measured using established caregiver burden instruments such as the ZBI and Neuropsychiatric Inventory (NPI). Further to explore how reducing pain, and therefore neuropsychiatric symptoms associated with increased caregiver burden impacts their delivery of care and their health and well-being, and the financial burden for caregivers as well as health care systems. This is especially relevant considering individual caregivers' financial strain and incapacity to work, as well as issues with worker's compensation claims, absenteeism, and staff retention for health care providers as a result of an increased caregiver burden (40–43).

## Conclusions

The results of this study demonstrated a clear relationship between the presence of resident experienced pain-related neuropsychiatric symptoms and caregiver burden, and that caregiver burden may be exacerbated by higher levels of pain. However, given that these indicators are embedded within the PainChek® pain assessment tool, these findings should be interpreted as reflecting co-occurring symptom patterns rather than independent predictors of pain or burden. These findings emphasize the need to educate caregivers, both professional and non-professional (i.e., family carers and others), on the importance of pain as a contributor to these symptoms and hence the burden of delivering care to people living with dementia. Further, our findings highlight the need to assess pain which can be challenging in people who cannot self-report, and the importance of ensuring adequate pain management as a means of reducing caregiver burden. This contributes towards a better quality of life for those living with dementia and those who care for them. The findings reinforce the clinical importance of recognising and managing pain-related neuropsychiatric symptoms as a strategy to support both people living with dementia and their caregivers.

## Data Availability

Deidentified data that does not violate confidentiality can be made available with the approval of PainChek Ltd, upon reasonable request from the date of publication and can only be used for research purposes, and not for product related work and cannot be shared with a third party, Further inquiries can be directed to the corresponding author.
